# Bioremediation of p-Nitrophenol by *Pseudomonas putida* 1274 strain

**DOI:** 10.1186/2052-336X-12-53

**Published:** 2014-02-28

**Authors:** Melvin S Samuel, Akella Sivaramakrishna, Alka Mehta

**Affiliations:** 1 School of Biosciences and Technology, VIT University, Vellore 632014, Tamil Nadu, India; 2 School of Advance Sciences, VIT University, Vellore 632014, Tamil Nadu, India

**Keywords:** p-Nitrophenol, *Pseudomonas putida*, Hydroquinone, Biodegradation, Bioremediation

## Abstract

**Background:**

p-Nitrophenol (PNP) occurs as contaminants of industrial effluents and it is the most important environmental pollutant and causes significant health and environmental risks, because it is toxic to many living organisms. Nevertheless, the information regarding PNP degradation pathways and their enzymes remain limited.

**Objective:**

To evaluate the efficacy of the *Pseudomonas Putida* 1274 for removal of PNP.

**Methods:**

*P. putida* MTCC 1274 was obtained from MTCC Chandigarh, India and cultured in the minimal medium in the presence of PNP. PNP degradation efficiency was compared under different pH and temperature ranges. The degraded product was isolated and analyzed with different chromatographic and spectroscopic techniques.

**Results:**

*P. putida* 1274 shows good growth and PNP degradation at 37°C in neutral pH. Acidic and alkali pH retarded the growth of *P. putida* as well as the PNP degradation. On the basis of specialized techniques, hydroquinone was identified as major degraded product. The pathway was identified for the biodegradation of PNP. It involved initial removal of the nitrate group and formation of hydroquinone as one of the intermediates.

**Conclusion:**

Our results suggested that *P. putida* 1274 strain would be a suitable aspirant for bioremediation of nitro-aromatic compounds contaminated sites in the environment.

## Introduction

The wide use of nitro-aromatics, as synthetic intermediate in the manufacture of pharmaceuticals, pigments, dyes, plastics, pesticides and fungicidal agents, explosives and industrial solvents [1], leads to accumulation of nitrophenols. p-Nitrophenol is a chemical compound that has a hydroxyl group and a nitro group [2,3]. These groups are attached to a benzene ring relatively in para position. PNP is an important intermediate in the manufacturing of azo dyes and a number of pesticides such as parathion, fluorodifen, nitrofen, bifenox, *p*-aminosalicylic acid and *p*-acetaminophenol [4]. PNP occur as contaminants of industrial effluents, soil and groundwater [5]. It is probably the most important among the mononitrophenols in terms of the quantities used and potential environmental contamination [6]. It has been classified as a priority pollutant by the United States Environmental Protection Agency, which recommends restricting PNP concentrations in natural waters, MCL (Maximum Contaminant Level) of 1 μg/L for phenols in drinking water [7]. It readily breaks down in surface water but takes a long time in deep soil and in groundwater. PNP poses significant health and environmental risks, because it is toxic to many living organisms and it may accumulate in the food chain. Therefore, rapid removal and detoxification of PNP is necessary [8,9]. Removal of PNP is achieved by physical, chemical or biological treatment processes and a variety of combinations of the treatment processes. Among the major physical treatment processes for PNP removal are adsorption, ultrasonic irradiation and microwave assisted oxidation. These physical methods like membrane-filtration is also associated with the disadvantage of membrane fouling and cost of periodic replacement [10,11]. Chemical methods of phenol abatement like coagulation, flocculation, precipitation-flocculation with Fe (II)/Ca(OH)2, electrokinetic coagulation, conventional oxidation by oxidizing agents are often expensive and although phenols are removed, accumulation of concentrated sludge creates a disposal problem. Secondary pollution problem may also arise due to excessive chemical use. The application of chemical treatment process for PNP removal is limited and only electrochemical oxidation has been recognized so far. However, the combinations of physical and chemical treatment processes are much prevalent. Biodegradation techniques by microorganism are preferable due to their economic advantage and the possibility of complete mineralization [12-18]. Studies carried out for the bioremediation of PNP showed that several bacterial species are capable of degrading PNP. Scientists have found that PNP can be utilized as sole carbon and energy source by Arthrobacter [19-21], Bacillus [15], Burkholderia [21], Pseudomonas [3,17,22-25], Alcaligenes [26] and Rhodococcus [5,27]. This study was carried out in search of more efficient *P. putida* strain for the bioremediation of PNP. The *P. putida* 1274 strain was used first time in this study. Since *P. putida* 1274 used in the present study has been isolated from soil near petrol bunk (South India), this strain may tolerate high concentrations of organic molecules and might be able to metabolize them. In our earlier studies we found that this *P. putida* 1274 strain can degrade aflatoxin B_1_ effectively [28], this aflatoxin B_1_ is an aromatic compound, the molecular structure is made up of di-furan coumarin ring and lactone portion [29], hence *p. putida* 1274 is also tested for the degradation of PNP. PNP degradation pathway was also analyzed and the major degradation product was identified using analytical techniques like UV spectroscopy, IR, GC-MS and HPLC. The formation of non-polar compounds during bioremediation was studied.

## Materials

### Preparation of medium and culture condition

*Pseudomonas putida* MTCC 1274 was obtained from MTCC Chandigarh, India and maintained on Nutrient agar medium (HiMedia Laboratories Pvt. Ltd, India) at 4°C. Growth studies were performed on Mineral salt glucose medium (MSG) [22].

### Culture conditions

Minimal salt glucose medium (MSG, pH 7.0) containing (g/L) Glucose - 20; K_2_HPO_4_ - 0.65; KH_2_PO_4_ - 0.2; MgSO_4_.7H_2_O - 0.09 and FeSO_4_.7H_2_O - 0.01. To the MSG broth PNP was added at the concentration of 50 μg/mL. PNP was purchased from Sigma-Aldrich (St Louis, MO, USA) solution was made in sterilized distilled water at the concentration of 1 mg/mL.

### Growth studies of *P. putida* strain 1274 in the presence and absence of PNP

The growth profile of the *P. putida* strain 1274 was carried in the MSG medium up to 24 h; every 3 h interval observations were taken. For this 20 mL of MSG broth with PNP (50 μg/mL) and without PNP was inoculated with Pseudomonas culture (100 μL of 0.5 OD at 600 nm) and incubated at 37°C temperature on shaker at 120 rpm [30]. Every 3 h growth of *P. putida* strains was determined by taking the absorbance at 600 nm in spectrophotometer (UV 1800, Shimadzu, Japan). The growth curve was made by plotting the Optical density (600 nm) of the culture against the time. Survival of pseudomonas in the presence of PNP was monitored by counting CFU (Colony forming unit) on the MSG agar medium. The Pseudomonas cultures were grown in the presence of PNP as mentioned above and every 6 h, 1 mL of the culture was withdrawn for CFU counting. The culture was diluted to 10^-4^ dilutions and 100 μL was inoculated on the MSG agar plate by spread plate method. Plates were incubated at 37°C and colonies formed were counted after 24 h of incubation. The control was maintained simultaneously in the absence of PNP.

### Degradation studies

To study the PNP degradation, cells were separated by centrifugation and the supernatant was extracted with twice the volume of diethyl ether and chloroform. Extracted solvents were evaporated to dryness at 37°C and re-dissolved in methanol for further analysis. Thin layer chromatography (TLC): The above crude extracts were applied to the TLC plate along with a standard PNP for qualitative analysis. Ten microlitre of the extract was applied on activated TLC plate (Silica gel 60 F254, Macherey-Nagel, Germany). Toluene: ethyl acetate: acetic acid (12:6:1, v/v) was used as solvent system [30]. After drying the plates were exposed to iodine vapour to check PNP and treated sample detection. The retention factor values of the treated sample were compared with the control.

PNP and treated samples were monitored by High Performance liquid chromatography (HPLC) (HPLC, Waters 1525). HPLC was carried out for di-ethyl ether and chloroform extracts. The stationary phase was C18 Polaris column. A sample of 20 μL was injected, methanol: water (80:20, v/v) with 0.1% acetic acid was used as mobile phase at a flow rate of 1 mL/min. UV-detector at 210 and 310 nm was used to monitor the degradation of PNP.

### Analytical studies for degraded compound

#### UV spectrophotometry

Wavelength scan was performed for the PNP and Pseudomonas treated samples of PNP using UV spectrophotometer from 200–800 nm. Similarly the wave length scan was performed for hydroquinone. The presence of PNP and hydroquinone was assessed by measuring the UV absorbance at 310 and 210 nm respectively.

#### Fourier transform infrared (FT- IR)

IR analysis was carried out with a KBr pellet using an IR spectrophotometer (IR Affinity -1, Shimadzu, Japan).

#### Gas chromatography (GCMS)

The control PNP sample along with the Pseudomons treated samples extracted with diethyl ether and chloroform were applied to GC-MS analysis. Thermo GC - trace ultra version: 5.0, thermo ms DSQ II, column: db35 - ms capillary standard non – polar, dimension: 30 Mts, ID: 0.25 mm, FILM: 0.25 μm, Carrier gas: Helium, flow rate: 1.0 mL/min, temperature program: oven temperature 40oC was raised to 270°C at 8°C/min. Injection volume 1 μL. Spectra system is UV6000LP ultraviolet (UV) detector.

### Qualitative analysis

#### Brown ring test

Iron sulphate and concentrated sulfuric acid was added slowly to the control and treated sample. Formation of brown ring at the junction of two layers confirms the presence of nitrate.

#### Mulliken and Barker’s reaction

To the control and treated sample, ethanol, few drops of calcium chloride, zinc dust was added, and boiled for 5 min. The sample was further filtered and to the filtrate tollens reagent was added. A bright silver mirror or black precipitate is formed. Confirms the presence of nitro group.

## Results

### Cell growth and PNP biodegradation

**Figure 1:**
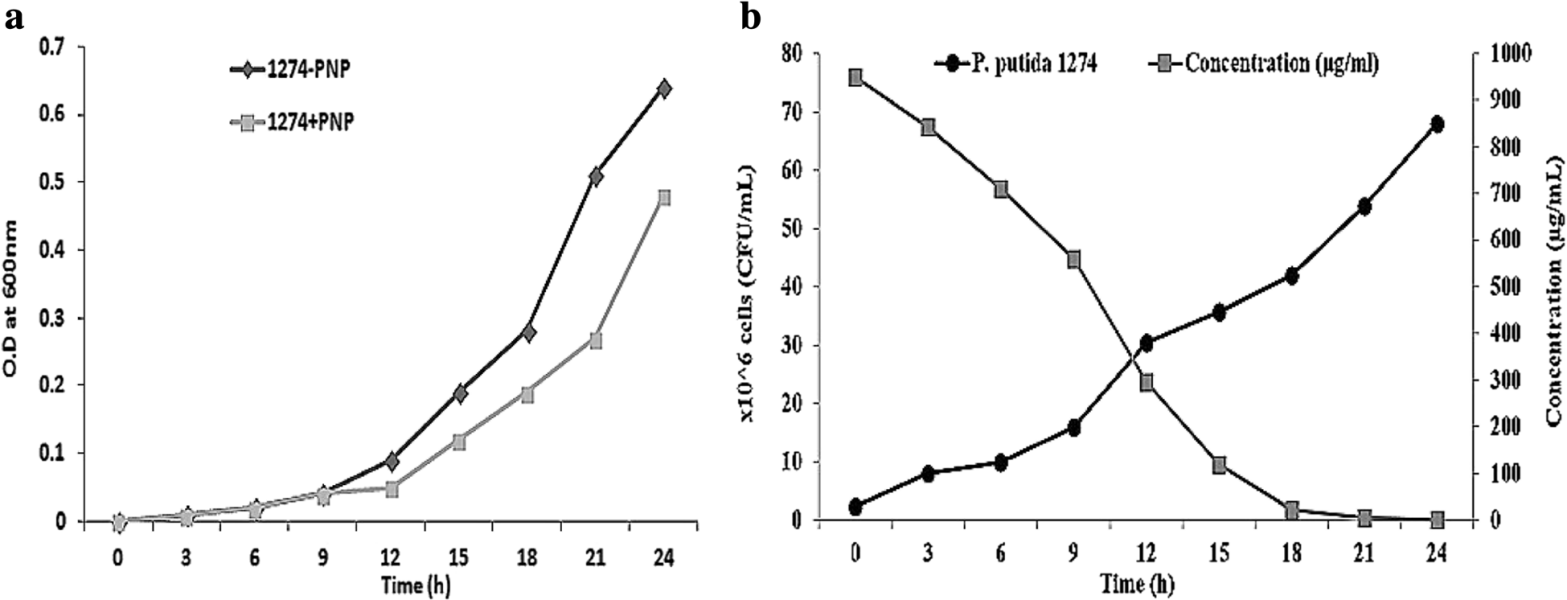
**Growth profile of *P. putida *** **strain (1274); a. in presence and absence of PNP. b. Growth of the *P. putida *** **1274 in PNP and depletion of PNP.**

**Figure 2:**
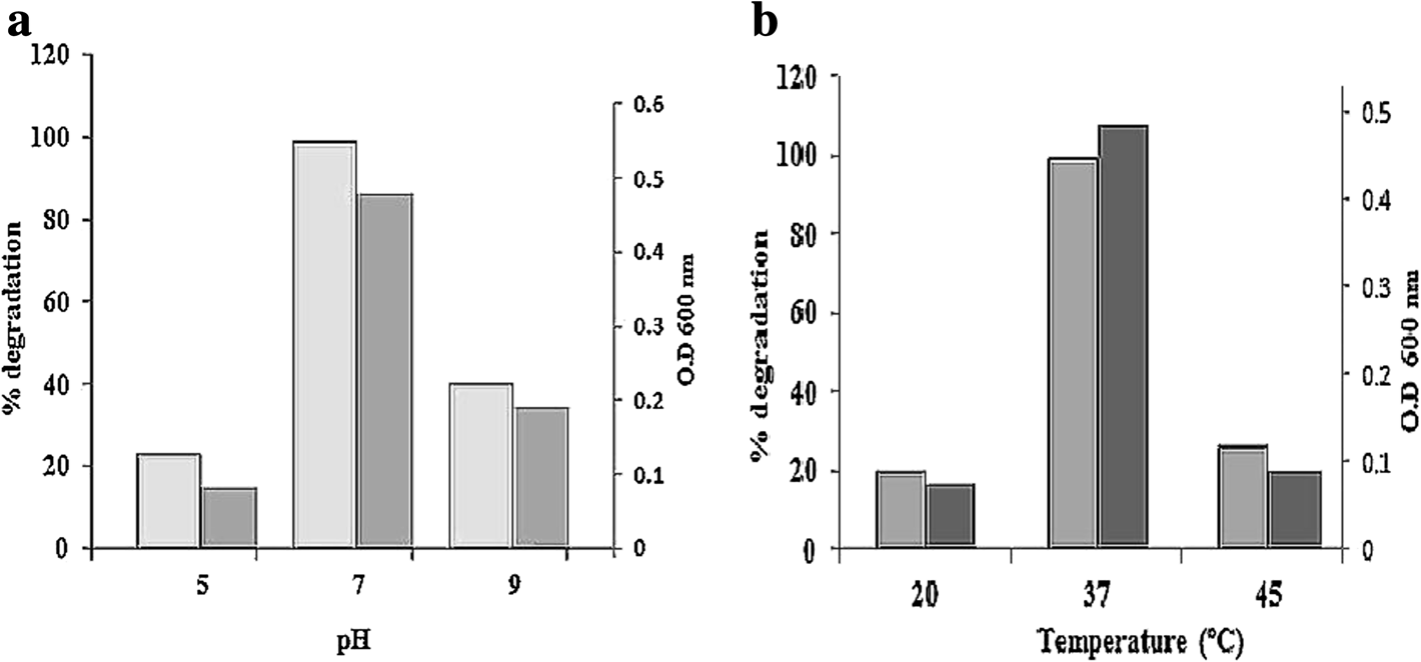
**Degradation of PNP by *P. putida*; a. at various pH; b. at various temperature.**

The growth studies showed that Pseudomonas after a lag phase of about 6 h grew fast and remain in exponential phase up to 24 h in the MSG medium. In the presence of PNP (50 μg/mL) in the MSG medium, the growth was retarded and optical density reached maximum 0.45 in 24 h. (Figure 1a). PNP at a concentration of 50 μg/mL was rapidly degraded in 24 h, associated with bacterial growth (Figure 1b); this might be due to the utilization of nitrate by the strain as a nitrogen source [31,32]. No degradation was observed in uninoculated controls. The effect of pH and temperature on the PNP degradation was investigated. The PNP degradation was studied at pH 5.0, 7.0 and 9.0. As well in different temperature ranges from 20°C, 37°C and 45°C. At pH 7 and 37°C, *P. putida* culture (O.D 600 nm - 0.48) was found to be most suitable for and displayed increase in PNP removal is shown in Figure 2a and b. From these result it can be inferred that the degradation of PNP has good correlation with the bacterial growth. Same time at lower pH, the phenol degradation is reduced in biochemical reaction and higher temperatures seemed to have a negative effect on the activity of the bacteria in laboratory condition and hence slowed down its biodegradation capabilities. It may have harmful effect on the bacterial culture, which is the main step in the biological degradation process [30].

### Degradation studies

**Figure 3:**
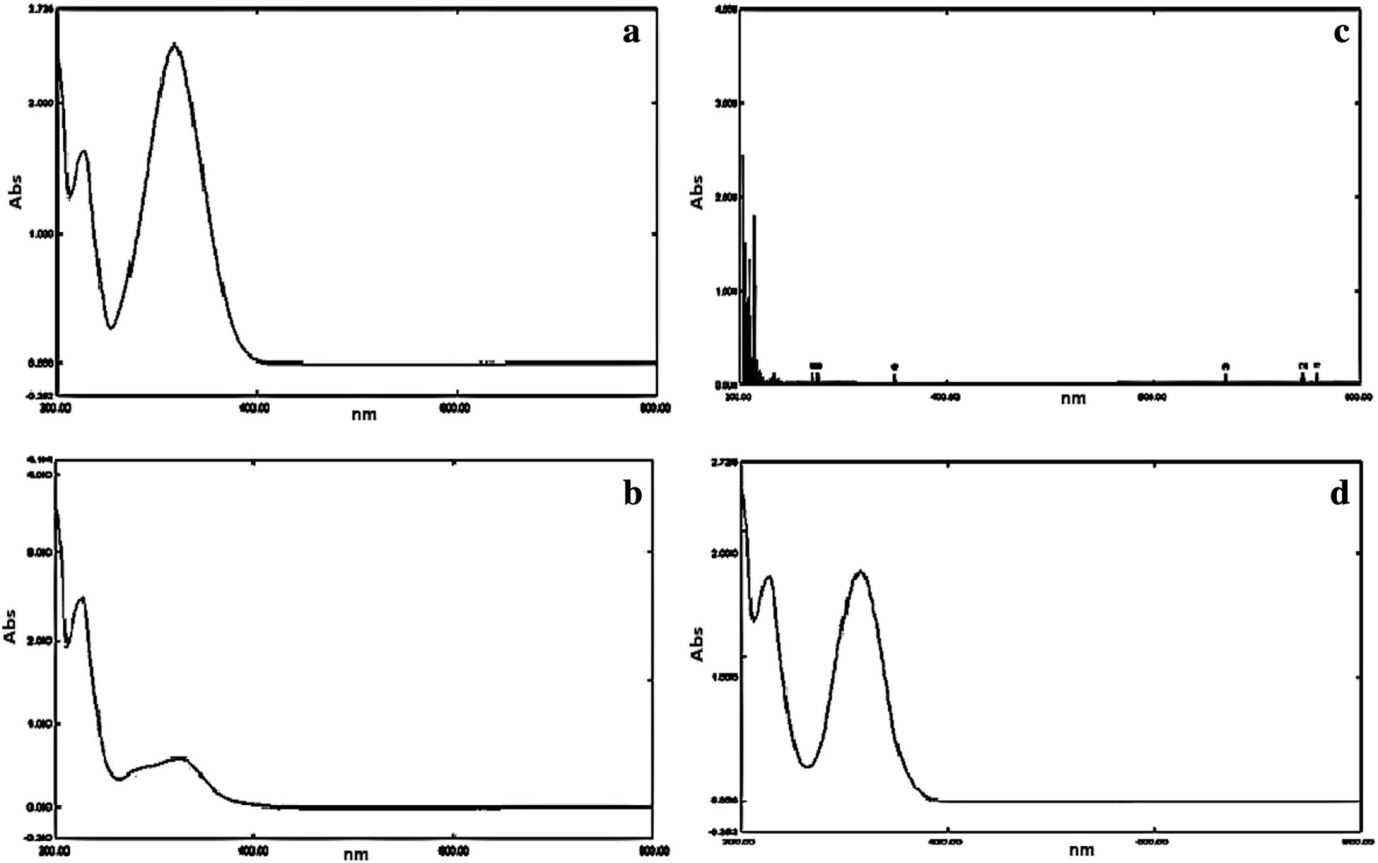
**UV-Visible spectrum of the PNP incubated with and without *P. putida *for 24 h. a**. Standard PNP (3 μg/mL); **b**. diethyl ether extract of treated PNP sample; **c**. chloroform extract of treated PNP sample; **d**. standard hydroquinone (3 μg/mL).

**Figure 4:**
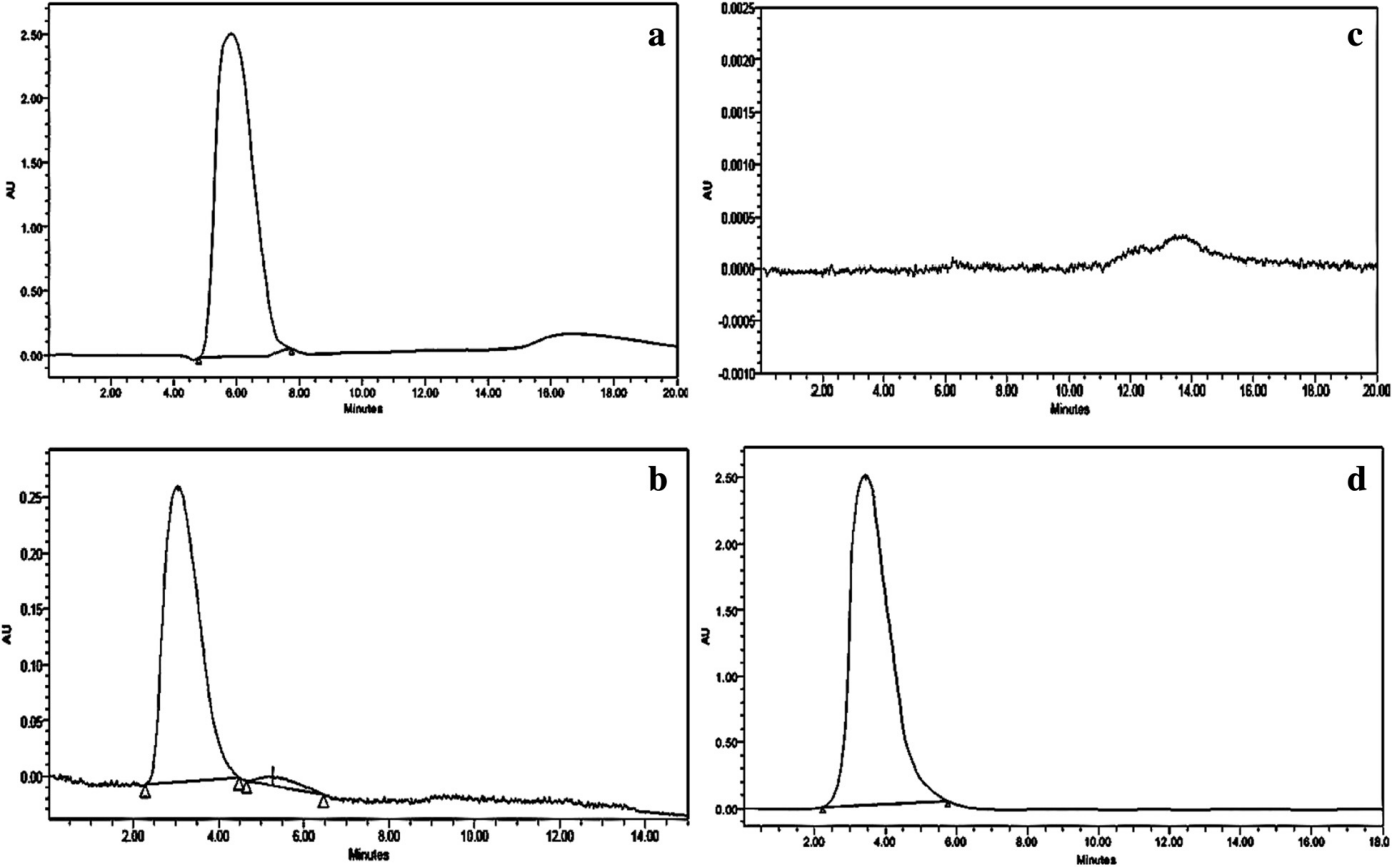
**HPLC chromatogram of the PNP incubated with and without *P. putida *for 24 h. a**. The chromatogram of standard PNP; **b**. diethyl ether extract of treated PNP sample; **c**. chloroform extract of treated PNP sample; **d**. the chromatogram of standard hydroquinone.

**Figure 5:**
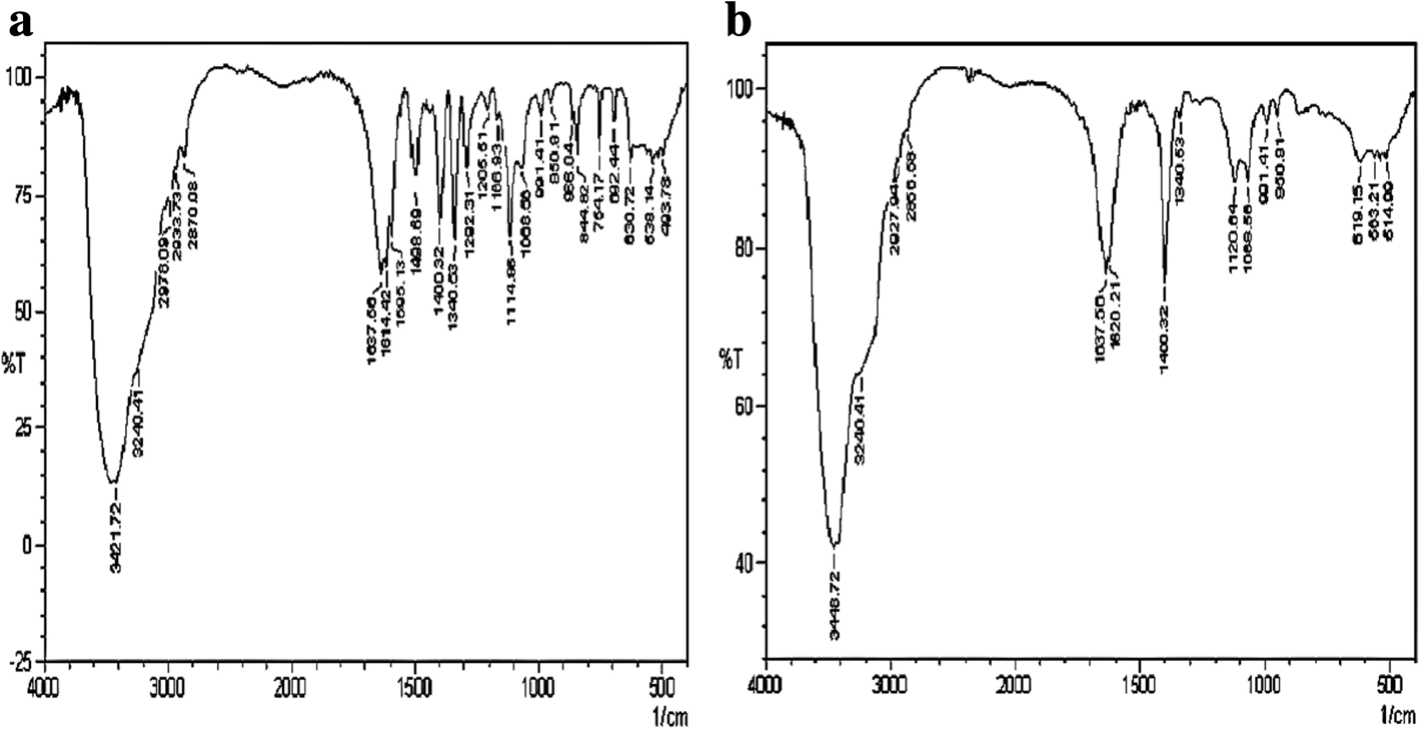
**Infrared spectrum of the PNP incubated with and without *P. putida *for 24 h. a**. PNP standard; **b**. diethyl ether extract of treated PNP sample.

**Figure 6:**
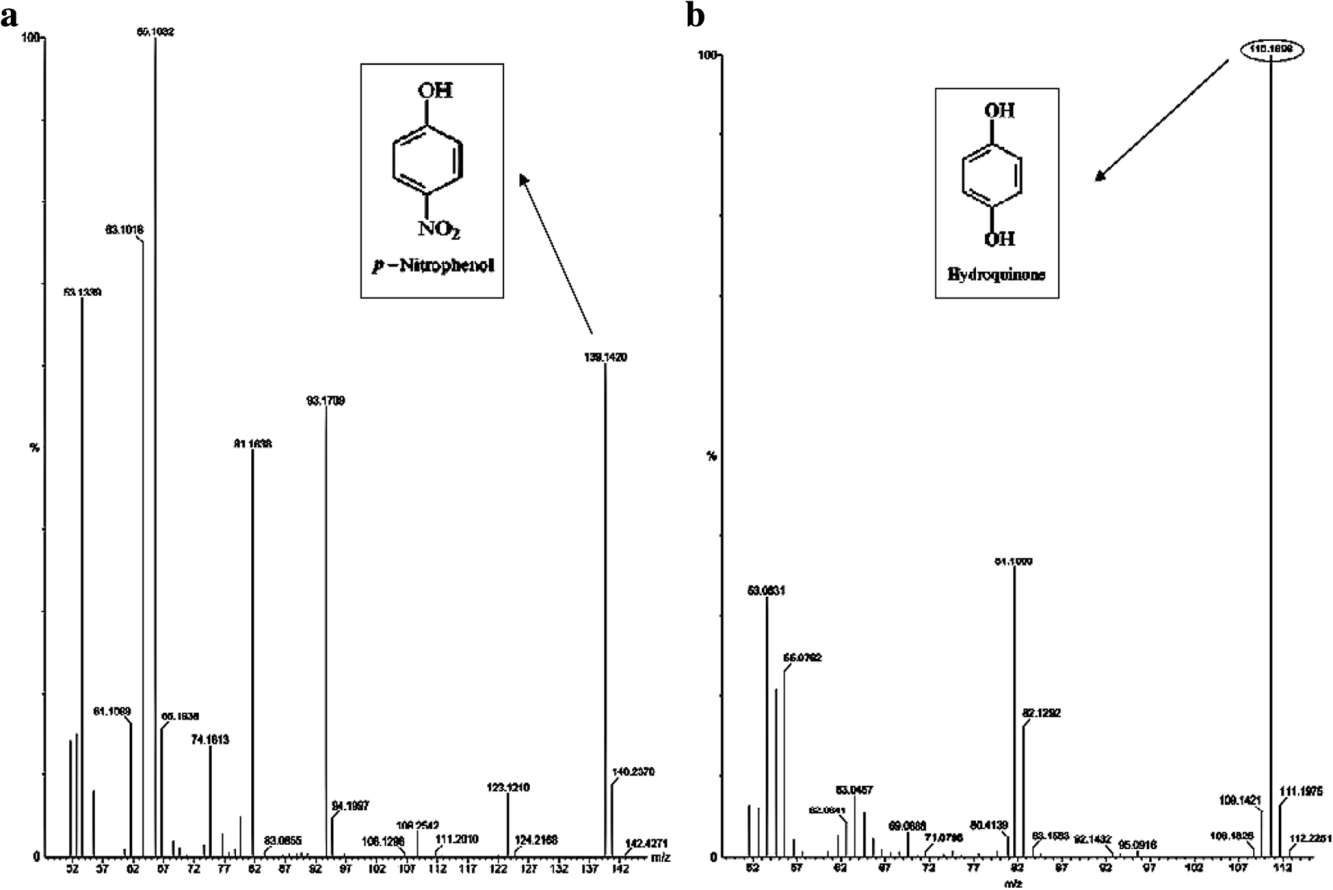
**GC-MS of the PNP incubated with and without *P. putida *for 24 h. a**. PNP standard; **b**. diethyl ether extract of treated PNP sample.

**Figure 7:**
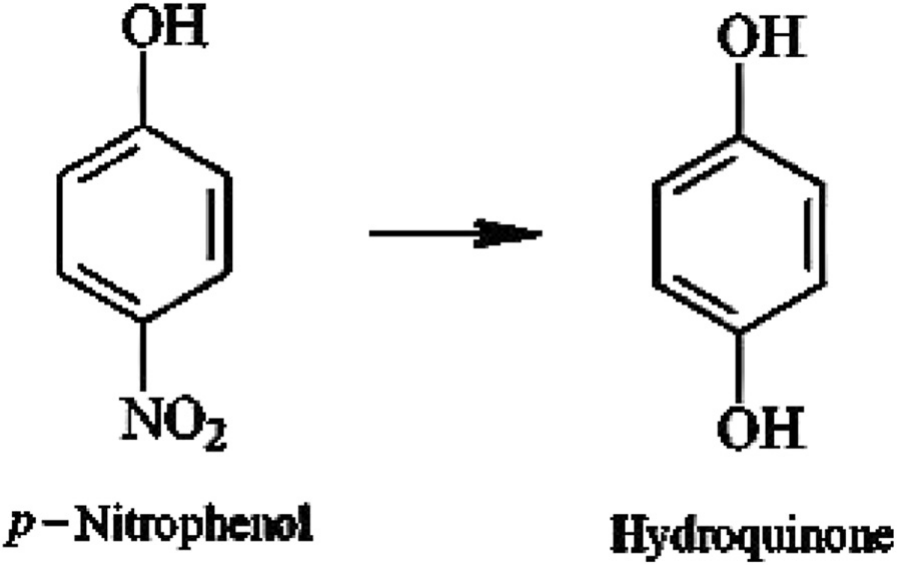
**Proposed scheme of PNP degradation.** Based on the UV–spectrum (Figure 3 a, b, c and d), HPLC (Figure 4 a, b, c and d), FT-IR (Figure 5 a and b) and GC-MS (Figure 6 a, and b) (Table 1).

PNP in the presence of *P. putida* was degraded as observed from TLC analysis, the variation in the retention time of the control and the sample indicated the degradation of PNP and exhibited the presence of new spot. The diethyl ether extract from the uninoculated sample showed the PNP spot at R_f_ 0.75 and Pseudomonas inoculated sample showed the degraded PNP spot at R_f_ 0.6. The UV-Visible absorption spectrum of PNP, treated sample extracted with diethyl ether and hydroquinone showed the λ_max_ at 310 nm and 210 nm respectively (Figure 3 a, b and d). Whereas chloroform extract did not show any absorption peak (Figure 3c). Further studies of the degraded product were carried out at 210 nm and checked with the standard hydroquinone (Figure 3d). The HPLC chromatogram at 210 nm confirmed that PNP peak eluting at 5.75 min (Figure 4a) disappeared in the treated sample extracted with diethyl ether. Similarly peaks were noted at 3.06 min of elution for the treated sample (Figure 4b) at 210 nm. Whereas the chloroform extract did not show any absorption peak (Figure 4c). The hydroquinone peak was also noted at 3.306 min (Figure 4d). FT-IR spectrum showed the absorption peak at 1290 cm^-1^, 1330 cm^-1^, 1595 cm^-1^ and 1498 cm^-1^ in PNP standard disappeared after the treatment (Figure 5a and b). In the analysis by GC-MS, PNP standard showed characteristic fragmentation ions with m/z 139, 123, 108, 93, 81 and 65 in the MS spectrum and intense peak was observed at m/z = 139 (Figure 6a). However, the distinct PNP ion at m/z =139 disappeared when the medium was supplemented with the *P. putida* culture for 24 h and showed fragment ions with m/z 110, 109, 82, 81, 63, 55and 53 in the MS spectrum and intense peak was observed at m/z =110 (Figure 6b). In qualitative test, the brown ring, Mulliken and Barker’s reaction proved the absence of nitrate group in the degraded sample (Table 1). The degraded compound was identified based on the UV– spectrophotometer, HPLC, FT-IR, GC-MS and qualitative chemical results as hydroquinone (Figure 7). The current study confirmed PNP biotransformation by *P. putida*.

**Table 1:** Qualitative chemical test for standard PNP and treated sample

**Qualitative test**	**Control PNP**	**Treated sample**
Brown ring	Positive	Negative
Mulliken and Barker’s	Positive	Negative

## Discussion

PNP has been reported to be highly toxic to most of the microorganisms. The toxicity of higher concentrations of nitro-aromatics limits its degradation by microbes. Therefore, degradation of PNP is mostly studied at lower concentrations. Several PNP-degrading microorganisms such as *Ochrobactrum, Moraxella, Nocardia, Arthrobacter, Ralstonia* and *Pseudomonas* can metabolize PNP by converting their nitro-groups as nitrites and utilize PNP as a source of carbon and nitrogen for growth at low concentrations (20–150 mg/L). Biodegradation of PNP by aforementioned microorganisms at high concentrations was rarely reported [1]. *Pseudomonas aeruginosa* HS-D38 could efficiently degrade PNP and used it as the sole carbon, nitrogen and energy source. *Arthrobacter protophormiae* RKJ100 is used in soils spiked with different concentrations of PNP compound and was observed that lower concentrations of PNP were efficiently degraded but not the high concentrations. This probably due to the toxic effects exerted by PNP on the organisms [33].

Present study demonstrates that *P. putida* 1274 can grow in presence of PNP, and within about 24 h it can completely degrade 50 μg/ mL PNP. Our results of PNP degradation at varying pH and temperature conditions suggest that a neutral pH and 37°C condition is vital in achieving high PNP degradation by *P. putida*. It was suggested that toxicity of PNP increases with a decrease in pH [30]. TLC analysis revealed disappearance of PNP accompanied by appearance of metabolite at R_f_ 0.6. In UV-Spectrophotometry control and experimental results showed no reduction in the PNP level (Figure 3a) where as a significant reduction in PNP concentration was observed upon treatment with Pseudomonas culture. The absorption spectra obtained during the degradation of PNP are shown in Figure 3b. This figure shows that the characteristic absorption of PNP disappeared and implies that PNP has been effectively removed from the medium. As the incubation time proceeded, the media changed from yellow to colorless, which suggested that there were some intermediates produced in the degradation of PNP. The removal of PNP may be attributed to the formation of hydroquinone by hydroxylation of benzene ring in the PNP molecule [20,34]. The HPLC analysis, also showed a peak at 3.06 min, which corresponds exactly to the standard hydroquinone (Figure 4b). This is a known intermediate in the biodegradation of PNP pathway [24,34]. We have checked for the non-polar degraded products simultaneously with PNP degradation to hydroquinone. The HPLC analysis of the chloroform extract of *P. putida* treated PNP shows absence of absorption peak indicating the absence of non-polar compound (Figure 4c). FT-IR spectrum of the major degraded product isolated from culture treatment clearly revealed the significant changes from the vibrational frequencies of the standard PNP. The absorption peak at 1290 cm^-1^, 1330 cm^-1^, 1595 cm^-1^ and 1498 cm^-1^ in PNP standard disappeared after the treatment, which indicates that the nitrogen group attached to the benzene ring was removed and followed by addition of oxygen group to the benzene structure which was confirmed by the presence of higher absorption peak at 3448 cm^-1^. GC-MS analysis of the standard PNP shows an intense peak at 139 m/z whereas the degraded compound showed the intense peak at m/z 110, it might be formed by the loss of NO group from the nitrobenzene group of PNP (Table 1). Further, the qualitative test confirmed the removal of nitrate group from the hydroquinone by brown ring test and Mulliken and Barker’s test, which did not give positive result for the treated sample. Two major initial degradation pathways of PNP by various microbes have been characterized. One is called hydroquinone pathway in which PNP is converted to maleylacetate via hydroquinone [21], another explains that PNP is converted via 4-nitrocatechol to 1,2,4-benzenetriol pathway [25]. The acute toxicity of PNP and hydroquinone was determined using a pulse-exposure (PE) testing protocol using larval American flag fish (*Jordanella floridae*) and PE LC20 (mg/L) values were hydroquinone, 0.13 and p-nitrophenol, 0.81 respectively, which inferred the toxicity of hydroqinone is comparatively lesser than PNP [35]. Our results indicated that *P. putida* 1274 degraded PNP as high as 50 μg/ mL and converted it to hydroquinone. It might be due to the hydroxylation of the ring at the para position of PNP that leads to hydroquinone formation. Thereby the bio-degradation of PNP to hydroquinone was confirmed by the above mentioned results.

Following the recent studies, biodegradation of PNP by immobilized Rhodococcus sp. Strain Y-1 was achieved in 48 h [5,27]. In soil samples, 100 mg kg^-1^ of PNP and 4-CP in mixtures were removed by strain LZ-1 (10^6^ cells g-1) within 14 and 16 days [25]. *P. putida* has been documented to be PNP-tolerant, and in about 72 h can completely degrade 500 ppm PNP, a concentration proven to be toxic to most of the microorganisms [30]. In our experiment we got complete degradation of PNP within this 18–24 h time, which shows that this *P. putida* 1274 is more efficient. Jain et al. [19] reported that in the presence of external carbon source the PNP degradation has been inhibited in Arthrobacter sp. Similarly the addition of glucose decreased the rate of PNP degradation even if increased cell growth occurred [35]. However in the present study conversion of PNP to hydroquinone occurred within 24 h of incubation before the exhaustion of glucose from the medium. This clearly indicates that in the presence of preferred carbon source (glucose) the ability of bioconversion of PNP to hydroquinone was not impaired in the Pseudomonas strain used in present study.

## Conclusion

This work reports that *P. putida* 1274 is able to degrade PNP. The major degraded compound was identified as hydroquinone based on the data obtained from TLC, HPLC, UV-Spectrometry, GC-MS, FT-IR and qualitative test. Due to strong degradation ability in less incubation time and adaptability to temperature variation, strain *P. putida* 1274 is a promising aspirant for the bioremediation of toxic phenolic compounds from the environment.

## Competing interests

The authors declare that they have no competing interests.

## Authors’ contributions

AM participated in the design of the study and supervised the work. MS did the analyses and AS interpreted the analyzed results. MS and AM wrote the initial draft and revised the paper critic ally for important intellectual content and compiled the work in accordance to journal format. All authors have read and approved the final manuscript.

## Authors’ information

1. Alka Mehta (AM) Professor, School of Biosciences and Technology, VIT University, Vellore, Tamil Nadu, India.

2. Melvin S. Samuel (MS) Research Scholar, School of Biosciences and Technology, VIT University, Vellore, Tamil Nadu, India.

3. Akella Sivaramkrishna (AS) Professor, School of Advance Sciences, VIT University, Vellore, Tamil Nadu, India.
